# Design and Development of a Low Cost, Non-Contact Infrared Thermometer with Range Compensation

**DOI:** 10.3390/s21113817

**Published:** 2021-05-31

**Authors:** Nicholas Wei-Jie Goh, Jun-Jie Poh, Joshua Yi Yeo, Benjamin Jun-Jie Aw, Szu Cheng Lai, Jayce Jian Wei Cheng, Christina Yuan Ling Tan, Samuel Ken-En Gan

**Affiliations:** 1 Antibody & Product Development Lab, EDDC, Agency for Science, Technology and Research (A*STAR), Singapore 138672, Singapore; nicholas_goh@alumni.sutd.edu.sg (N.W.-J.G.); anson_12@hotmail.com (J.-J.P.); joshua_yeo@eddc.a-star.edu.sg (J.Y.Y.); benjaminaw021@gmail.com (B.J.-J.A.); 2 Institute of Materials Research and Engineering, A*STAR, Singapore 138634, Singapore; sc-lai@imre.a-star.edu.sg (S.C.L.); jayce_cheng@imre.a-star.edu.sg (J.J.W.C.); yl-tan@imre.a-star.edu.sg (C.Y.L.T.); 3 Department of Psychology, James Cook University, Singapore 387380, Singapore

**Keywords:** infrared, Arduino, nano, contactless thermometer, HC-SR04, MLX90614

## Abstract

Fever is a common symptom of many infections, e.g., in the ongoing COVID-19 pandemic, keeping monitoring devices such as thermometers in constant demand. Recent technological advancements have made infrared (IR) thermometers the choice for contactless screening of multiple individuals. Yet, even so, the measurement accuracy of such thermometers is affected by many factors including the distance from the volunteers’ forehead, impurities (such as sweat), and the location measured on the volunteers’ forehead. To overcome these factors, we describe the assembly of an Arduino-based digital IR thermometer with distance correction using the MLX90614 IR thermometer and HC-SR04 ultrasonic sensors. Coupled with some analysis of these factors, we also found ways to programme compensation methods for the final assembled digital IR thermometer to provide more accurate readings and measurements.

## 1. Introduction

Early methods of measuring body core temperature utilizing contact mercury thermometers are replaced by the safer and more convenient electronic thermometers at the sublingual, armpits, ear canals, and in some rare occasions, the rectum and axillary for accuracy [1]. Many of these surface measurement sites, specifically the temporal and central forehead, reflect lower readings than internal sites such as the tympanic temperature readings, the current gold standard to represent the body core temperature [2], especially given the impracticality of rectal/anal temperature takings.

While screening for disease in the ongoing pandemic, rapid temperature measurements of many individuals quickly and safely without allowing the thermometer to be a vector of pathogen transfer are crucial, thus making contact infeasible, ruling out many of the above measurement sites. 

Infrared (IR) thermometers can fulfil this gap by measuring the surface temperature without direct contact, which is through detecting the amount of thermal or black-body radiation emitted by the object. Additionally, these thermometers are now commonly used in clinical practices [3], as well as routinely during the pandemic for self-monitoring and screening at the entrances of public places.

Typically, IR thermometer casings are manufactured by the expensive injection molding (due to mold production and tooling costs), producing significant waste material. The increased adoption of three-dimensional (3D) printing technology has revolutionized prototyping and reduced cost by shortening the lead time to manufacture with significantly less waste. Coupling with 3D printing, the use of ‘off-the-shelf’ microcontroller kits such as Arduino, Raspberry Pi, and Micro: bit can now allow novel electronic products to be cost-effectively assembled, even by non-engineers without specialized equipment. It is with such enabling technology that even home-made measurement devices can be made easily, e.g., spectrophotometers [4,5], including those for psychology research use [6]. 

While thermometers can be easily assembled, IR thermometers are often thought to be less reliable [7] than traditional contact thermometers. Non-contract infrared thermometers were previously reported to have a sensitivity between 4.0% to 89.6%, specificity between 75.4% to 99.6%, and a positive predictive value between 0.9% to 76.0% [8]. In fact, there are recommendations for its repeated measurements at hospital gantries [9], given that IR thermometers are highly prone to external interferences by surrounding temperatures, relative humidity [10], the site of measurement [11], and the presence of oil (sebum) and sweat on the forehead [12], as well as other factors in the immediate environment [13]. Apart from these innate factors, intrinsic human physiological factors such as fever [14] or exercise [15] can produce sweat to affect the measurements. With further confounding influence from the surrounding temperature and humidity that affect blood supply to the skin surface, which by generally lower than the expected body temperature [16,17], many IR thermometers, especially self-assembled ones can be inaccurate as they lack ambient temperature and distance sensors [7] for compensation. 

At the point of writing, many IR sensors have in-built radiation emitter and receiver devices [18] and can be used to provide reliable measurements at predetermined distances. Yet, the onus is still on the user to operate them correctly for accurate measurements.

To help alleviate the above problems, we describe the design and assembly of a low cost IR thermometer with distance and environmental temperature sensing capabilities to provide more accurate measurements. Experiments were conducted to validate compensation adjustments made in the algorithm, as well as the effects of measurements on different locations of the forehead. 

## 2. Materials and Methods

### 2.1. Components and Specifications

Arduino Nano (HWA YEH, Shenzhen, China), ultrasonic sensor (HC-SR04) [19], infrared sensor (MLX90614) [20], and OLED display (SSD1306) [21] were used. A factory calibrated MLX90614 infrared sensor, suitable for a wide range of temperatures between −40 to 85 °C for the ambient temperature and −70 to 1030 °C for the target object temperature, provided the average temperature of all objects within the field of view (FoV) of the sensor, as well as a standard accuracy of 0.5 °C around room temperature. The Ultrasonic Sensor HC-SR04 emits a 40 kHz ultrasound and computes the distance based on the time taken to detect the ultrasound wave reflected off an object. An SSD1306 organic light-emitting diode (OLED) display is incorporated to display the readings.

### 2.2. Assembly of Infrared Thermometer

The design and development of the thermometer in this project were adapted and modified from online instructions [22] and comprise two main aspects: (1) The physical form and mechanical assembly; (2) The electronic circuit, including the firmware.

#### 2.2.1. Physical Assembly 

**Figure 1:**
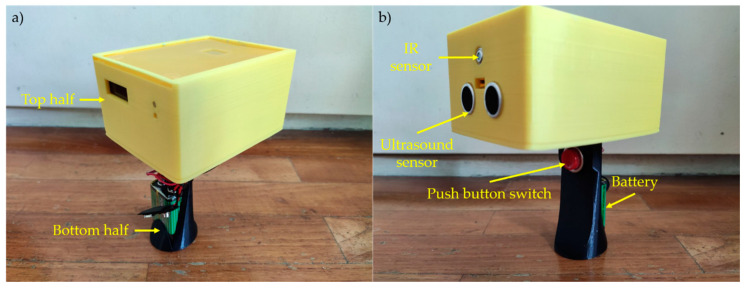
(**a**) Back view of prototype segmented into the top (yellow) and bottom (black) half with color differentiation for display purposes. (**b**) Front view of prototype displaying the IR sensor and ultrasound sensor in the top half. The bottom half of the device includes the push button switch and battery holder.

Figure 1a shows the physical design with the top half (Figure 1, in yellow) housing the components: IR sensor, ultrasound sensor, Arduino Nano, LED bulbs, and OLED display. The bottom half (Figure 1, in black) comprises the battery holder and a push button switch. The shell (top and bottom half) was 3D printed with a polylactic acid (PLA) filament (3D AURA Pte Ltd., Singapore) using a Prusa i3 Mk3 3D printer.

#### 2.2.2. Circuitry Design and Implementation

**Figure 2:**
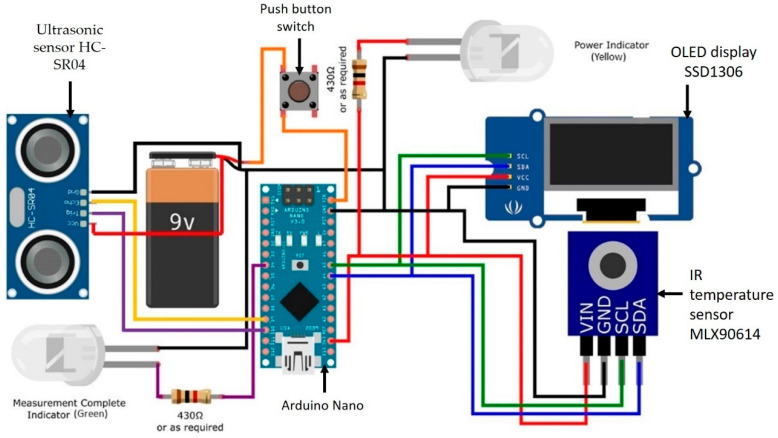
A circuit schematic of the assembled digital IR thermometer consisting of: Ultrasonic sensor HC-SR04, push button switch, Arduino Nano board, OLED display SSD1306, and IR temperature sensor MLX90614.

The Arduino Nano [23] microcontroller, in which the copper wires from the sensor pins of the ultrasonic sensor, IR sensor, OLED, and push button switch were soldered, are shown in Figure 2, with a 9 V battery supplying 3.3 to 5 V to the circuit (Figure 2).

### 2.3. Programming Arrangement

**Figure 3:**
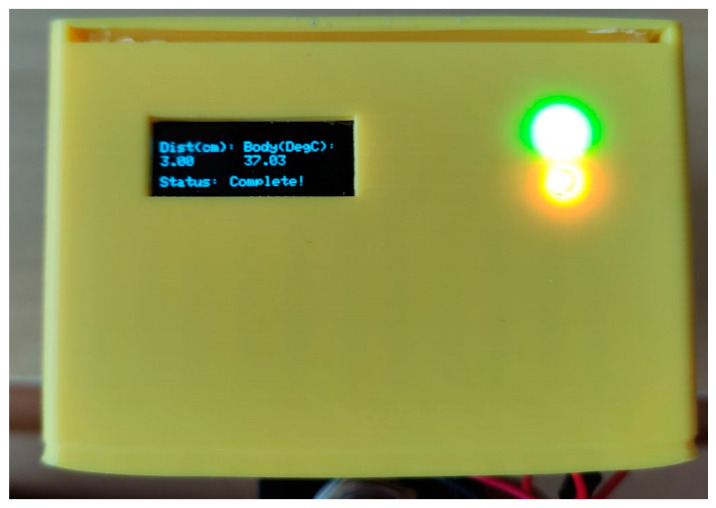
OLED display illustrating the distance to the target surface, measured surface temperature, and the measurement status.

The Arduino IDE 1.8.13 software is used to program the thermometer. Holding down the push button activates the measurement within 2–4 cm (the range recommended by sensor manufacturers) from the volunteers’ forehead. Five readings from the ambient and surface body temperature would be made and the average shown. Simultaneously, the ultrasonic sensor detects the distance from the target area and calculates the average of five readings. With the inputs of distance and temperature to the algorithm, the compensated reading from the algorithm would be displayed on the mono color, 128 × 64 pixels OLED module (Figure 3). 

The compensation adjustment equation was derived from the regression analysis of the relationship between the oral temperature values and the measured values. Compensated values by the IR thermometer were used as the dependent value, while the measurement values by the IR thermometer and ambient temperature were each viewed as independent variables. 

### 2.4. Sensor Calculations

#### 2.4.1. Infrared Sensor

The equations and calculations of the target temperature are based on a previous work by others [24]. For precise measurement of the absolute temperature of the target (T_k_), the device temperature (T_dev_) should be kept small with a stable ambient temperature (T_amb_). To compensate for the proximity effects, a distance-to-spot ratio (D/S ratio) is built in the algorithm. Specifically, the area measured increases as the distance increases. The selected IR temperature sensor has a field of view of 80 degrees. This translates to a D/S ratio of 1:1.68. Setting the average height of a human forehead of 58.3 mm as the constraint [25], the maximum horizontal distance that the IR temperature sensor can reliably measure the temperature of the target, is approximately 4 cm. Beyond this distance, flanking areas of the forehead would also be measured, affecting the accuracy. 

#### 2.4.2. Ultrasound Sensor

The ultrasound sensor consists of a transmitter sending an ultrasound wave and a receiver detecting the reflected wave by the targeted physical object. The time taken between the transmission and detected wave is registered for the calculation of the distance from the speed of ultrasound waves at 330 m/s by the Arduino Nano. 

#### 2.4.3. Experimental Setup

An ethical review and approval were waived for this study, due to the low risk and anonymized data collection under A*STAR IRB reference number 2021-006 for recruitment of five volunteers (aged 21 years and above) with anonymized informed consent. To ensure anonymity, a random volunteer ID between 1 to 1000 was generated for each volunteer entry. 

Three experiments were performed for this study. Each experiment was conducted in an air-conditioned environment of 20 to 22 °C for a relatively constant T_amb_. Volunteers were asked to avoid high intensity activities before the experiments, and temperature measurements were taken 5 min after acclimatization to the test environment. A sterilized Omron digital thermometer MC-343F was used to measure the oral temperatures, which was also set as the target point for compensation adjustments. Temperature measurements of the volunteers’ forehead were taken in triplicate experiments. 

In the first experiment, the IR thermometer was used to measure the volunteers’ forehead temperatures at three different locations (left, right, and center of forehead) at varying distances from the forehead (2–4 cm, at 0.5 cm intervals). The range of 2–4 cm is based on the minimum and maximum allowable horizontal distance that the IR temperature sensor can accurately measure (2–4 cm) as per the manufacturer’s recommendations. In the second experiment, water was sprayed on the volunteers’ forehead using a spray bottle to simulate wetness from sweat. 

The IR and oral temperature readings from the first experiment set were used for compensation adjustments and stored. Then, the calibrated IR thermometer was used to measure the center and lateral forehead temperatures of volunteers and test its performance and deviation from the oral temperature. Data from each experiment were plotted with the distance against the temperature. 

#### 2.4.4. Statistical Analysis

The mean and standard deviations of temperature readings were calculated and analyzed using Microsoft Excel (version 15.0) and expressed as a linear regression showing the R^2^ value. The one-way analysis of variance (ANOVA) followed by Dunnett’s multiple comparison tests were performed using the R software (version 3.6.2) [26] to compare the mean recorded temperatures of each volunteer relative to the control (oral temperature). Cronbach’s α was set at *p* < 0.05.

## 3. Results

### 3.1. Performance of IR Thermomter on the Forehead

**Figure 4:**
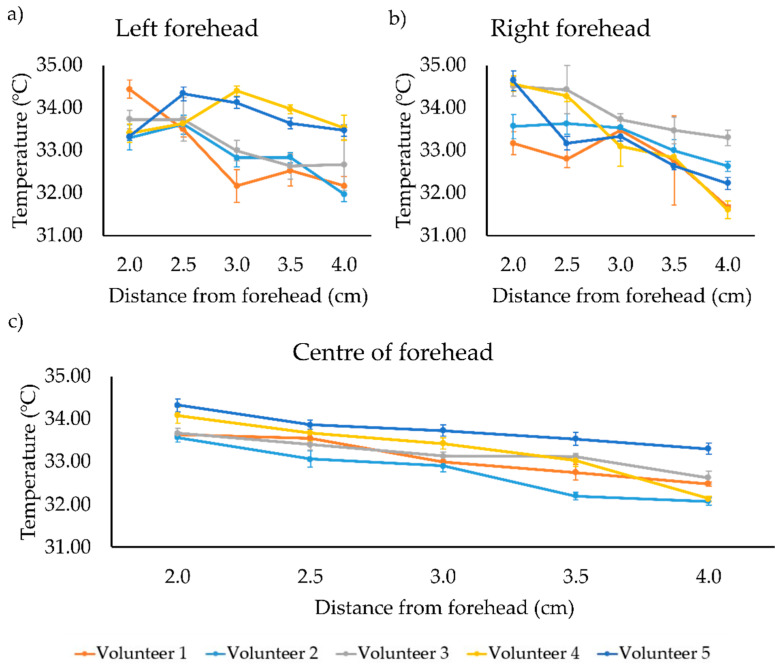
Measured forehead temperatures against the distance between 2–4 cm for each volunteer taken on the (**a**) left of the forehead, (**b**) right of the forehead, and (**c**) center of the forehead prior to the compensation adjustments. Error bars represent the standard deviation from triplicate experiments.

Prior to the compensation adjustments of the IR thermometer, an inverse correlation between the temperature readout and distance from the forehead was measured for all the five volunteers at all three forehead locations (left, right, and center, see Figure 4).

The variation of the mean temperature measurements between the center and lateral areas of the forehead were calculated to be between −0.69 to 0.55 °C (Table 1). The center is taken as the most accurate region for compensation, in view of the stronger correlative relationship between the recorded temperatures and distance.

### 3.2. Performance of IR Thermomter on the Wet Forehead

Variations in the mean temperature measurements between the wet and dry forehead ranged from 3.06 ± 0.00 to −2.85 ± 0.02 °C (Table 2 and Table S1). Generally, the mean wet forehead temperatures were lower than dry forehead temperatures, with very few exceptions. This finding confirms the concern where perspiration or wet weathers may result in false negatives of fever during screening. 

### 3.3. Compensation Adjustments and Testing of IR Thermometer

**Figure 5:**
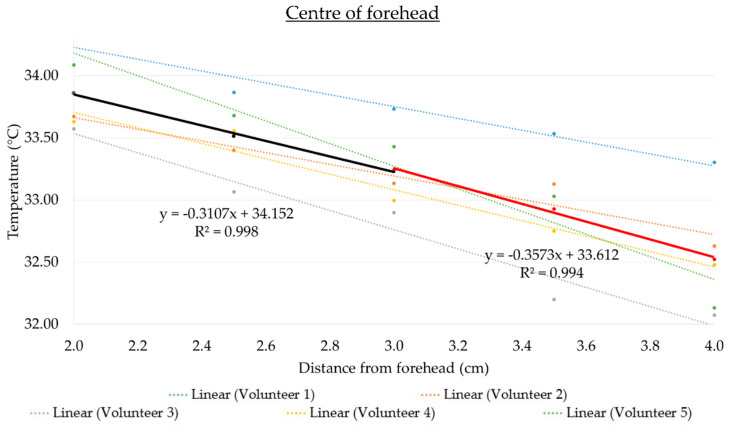
Combined trend lines for all the volunteers between 2–3 cm (in black) and between 3–4 cm (in red).

Linear regression models were applied on the measured readings for each volunteer. The compensation adjustments lines for the temperature (y) against the distance (x) over two different ranges (2–3 and 3–4 cm, respectively) were obtained, as shown in Figure 5. The data points between 2–3 cm are used to generate a best-fit straight-line equation y = −0.3107x + 34.152, with a coefficient of determination R^2^ = 0.998. Similarly, a best-fit straight-line equation of y = −0.3573x + 33.612 is obtained using the measured readings from 3–4 cm, with R^2^ = 0.994. With the R^2^ close to 1 in both linear regression models, the sensor used in this experiment is shown to have high reproducibility for measuring the temperature (based on the IR temperature sensor) and distance.

### 3.4. Test Performance of the Optimized IR Temperature System

The performance of our implemented control system with distance-sensing capabilities was tested factoring the gradient into the algorithm. For compensation, a fixed arbitrary number was added to the raw temperature outputs, given that this would differ across various sources of manufactured IR sensors. 

Following compensation adjustments, experiments were repeated with the compensation applied to the measurements. We found only the left forehead of Volunteer 4 to have significant differences (an average of 1.36 °C, *p* < 0.001) from the oral temperature using Dunnett’s post-hoc test. Across the five volunteers, the mean temperature measured at the center of the forehead (over the left and right) is closer to the mean oral temperature (Figure S1). Thus, subsequent measurements were performed based on the center of the forehead temperatures.

**Figure 6:**
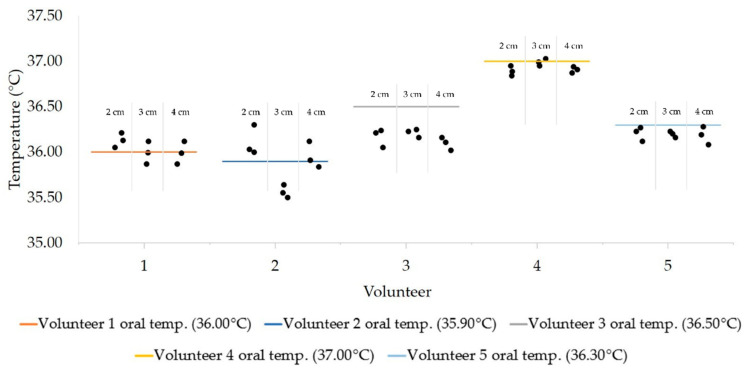
Recorded temperatures and the oral temperature for each volunteer. For each volunteer, the dots represent individual data points in the respective distance from the foreheads.

Based on the ANOVA results, there was no statistically significant difference in the temperature measurement from the center or lateral area of the forehead against the oral temperature (Figure S1). However, Dunnett’s post-hoc test showed that the *p*-value differences between the measurements from the center of the forehead to the control was higher than that of the measurement of lateral areas to the control across all volunteers. Between 2–4 cm, the measured temperatures were within a range of ±0.29°C of their oral temperature (Figure 6). Despite the presence of outliers, the recorded temperature displayed a standard deviation of ±0.3 °C (Volunteer 3) from the oral temperature.

## 4. Discussion

We set out to assemble and calibrate an Arduino-based thermometer capable of compensating for varying measurement distances from the forehead for a more accurate contactless body temperature measurement.

With the availability of off-the-shelf electronic sensors and microcontroller kits, it is possible for non-engineers to assemble their own devices to meet times of shortages such as that experienced by the authors when self-monitoring measures were implemented during the COVID-19 pandemic in 2020. While it will take a significant time for such self-assembled thermometers to be approved by regulatory bodies and pass the required calibrations as required for commercial devices, at times of extended shortages, such self-assembled devices allow an immediate patch solution also known in India as “Jugaad”. Yet, the potential problem arising from inaccurate measurements can have dire effects in infection control measures with false negatives, especially given that the forehead temperature rarely goes above 35 °C. In recognition of such effects, we have decided to release our results for the community to tweak their own assembled thermometers.

### 4.1. Assembly of the Arduino-Based Thermometer

We adopted Arduino for its ease of use and variety of sizes for assembly. While Scratch programming with Micro:bit [27] may be simpler in terms of programming, its larger size and pin connectivity pose a problem for small handheld thermometers. Size is also a concern for the more powerful alternative Raspberry Pi [28]. To balance these considerations, we adapted an online DIY thermometer assembly guide, using Arduino Nano to connect the available devices.

To reduce the measurement error caused by improper targeting at the forehead, we programmed the thermometer to take five readings and display the average. Similarly, the sensors allow for ambient temperature sensing, that together with our added ultrasound module for distance measurements, allow for compensations to be applied once the effects of some common parameters such as forehead location, wetness, and distance of measurement, are established.

### 4.2. Differences in Temperature between the Center and Lateral Areas of the Forehead

We first sought to investigate if there were differences on the surface temperature between the center and the lateral areas of the forehead. Differences between the center and lateral areas of the forehead were observed and persisted even after compensation adjustments (Figure S1). This difference in mean temperature is also attributed to the physiological phenomenon explained by the individual’s unique forehead temperature distribution as determined through the simulation software [29]. It should be noted that the IR sensor in our assembled thermometer is commonly used to measure the surface temperature of the skin and is unable to determine the muscle temperature near the superficial temporal artery, a major artery located beneath the vascular bed of the head’s skin [30]. While the differences between the forehead locations are small and perhaps inconsequential in differentiating high fevers above 38 °C, our studies show that using the IR thermometer for the center of the forehead is the most accurate and consistent. Given that this is a specific forehead locale variable, compensation mechanisms are not easily programmed into the device so the onus of accuracy falls upon the correct usage by the user. 

### 4.3. Wetness of Forehead

In the second experiment set, the mean wet forehead temperatures were typically lower than the dry forehead temperatures with exceptions. Consistent with a previous study where a high performance thermal imaging camera also detected a lower temperature at various parts of a perspiring body [24,31], temperature measurements taken with an IR thermometer on wet foreheads can be inaccurate. However, there are no compensation measures that we could incorporate since we could not incorporate wetness detection. Therefore, compliance to the correct practice of measuring the right target location and for the surface to be dry are required to avoid false negatives. 

### 4.4. Performance of Calibrated IR Thermometer

Our improved IR thermometer recorded an average temperature within ±0.29 °C of the volunteers’ oral temperature (Figure 6). As measurements for oral temperature are widely used to non-invasively measure the body temperature, measurements of the forehead temperature can be used to establish a threshold in the screening for fever. The IR thermometer obtained a better precision compared to the alternative and more expensive non-contact IR devices and was improved distance compensation. In a separate study, tympanic and forehead temperatures taken by a BRAUN IRT-3020 had an error range of ±0.286 and ±0.392 °C [24]. Another study showed tympanic and forehead temperature errors of ±0.37 and ±0.36 °C respectively [32]. However, other contact devices such as the rectal thermometer, with a small error range of ±0.05 °C [33] outperformed our improved thermometer. Still, our improved device costs only a fraction of commercial IR thermometers and was sufficiently accurate to be considered as a low cost alternative (~USD 7.50, USD 10) to the current entry level IR thermometers (>USD 80) in the market. This non-contact device is more convenient than the tympanic or rectal thermometers.

To improve accuracy, there are numerous established methods to calibrate IR thermometers [34,35]. For our purposes, distance compensation did improve accuracy as it did with others [32,35].

For clinical grade temperature measurements of the forehead lateral areas, contact type thermometers are recommended [36], where its sensing element, a thermistor comes in contact with the skin of the forehead or are inserted into the muscle. While contact type thermometers are recommended over non-contact types, it is not widely adopted since IR thermometers have greater sensitivity and specificity over contact temporal thermometers for temperatures >37.5 °C [37]. In working towards commercial use, it may be useful to use a second order equation for compensation adjustments from our two first-order equations, as well as to bear in mind the potential revisions on the International Temperature Scale of 1990 (ITS-90) for better reproducibility [38], along with the use of smart materials to make the thermometer casings [39].

## 5. Conclusions

Forehead temperature measurements using an IR thermometer play an important role of rapidly screening for fever to identify the infected individual. The performance and precision of an IR thermometer for forehead temperature screening were studied together with the design and implementation of an improved infrared temperature sensor-based system with distance sensing capabilities. 

While minimal, temperature differences between the center and lateral areas of the forehead highlight the importance of the user in targeting the right area, which is the center of the forehead. 

Additionally, we showed that perspiration and water on the forehead can cause a significant decrease in the detected temperature, but were unable to make programmed compensations. Therefore, it is necessary for the user to take precaution in ensuring that the forehead is dry and the skin surface temperature is restored before accurate measurements can be taken.

After implementation of a range sensor, the preliminary results show that our IR thermometer can achieve a more accurate forehead temperature measurement over a short distance range. This is achieved by obtaining an optimized algorithm from experimentally plotted linear regression lines. Moreover, it is observed that the measured temperatures were well within the ±0.29 °C variation of their oral temperature over the distance of 2–4 cm, achieving a similar performance to commercial thermometers. Therefore, it could be concluded that the designed control system has a high validity to measure human forehead temperatures within the compensated range. 

## Figures and Tables

**Table 1:** Variations in the forehead temperature measured from the center and lateral positions of the forehead of five volunteers using our IR thermometer prior to the compensation adjustments.

Volunteer	Temperature Difference between the Center of the Forehead and Left Forehead (°C)	Temperature Difference between the Center of the Forehead and Right Forehead (°C)
1	0.13 ± 0.44	0.31 ± 0.34
2	−0.16 ± 0.35	−0.51 ± 0.31
3	0.04 ± 0.27	−0.69 ± 0.27
4	−0.51 ± 0.34	0.00 ± 0.56
5	−0.03 ± 0.23	0.55 ± 0.40

**Table 2:** Difference between the dry and wet forehead temperatures on the respective locations from 2–4 cm. The positive value implies that the dry forehead registers a higher temperature.

Distance (cm)	Volunteer	Difference between the Dry and Wet (Dry-Wet) Forehead (°C)		
		Center	Left	Right
2.0	1	1.34 ± 0.01	−2.85 ± 0.02	1.57 ± 0.01
	2	1.85 ± 0.08	0.80 ± 0.07	2.35 ± 0.25
	3	2.49 ± 0.03	2.01 ± 0.06	0.34 ± 0.05
	4	1.89 ± 0.04	1.41 ± 0.02	0.96 ± 0.19
	5	1.03 ± 0.06	1.55 ± 0.07	−0.18 ± 0.22
2.5	1	1.69 ± 0.05	2.20 ± 0.01	2.81 ± 0.27
	2	1.58 ± 0.01	0.28 ± 0.01	0.26 ± 0.07
	3	2.07 ± 0.02	1.55 ± 0.01	−0.35 ± 0.18
	4	2.33 ± 0.05	1.34 ± 0.08	2.55 ± 0.17
	5	0.51 ± 0.89	0.72 ± 0.01	−0.24 ± 0.22
3.0	1	1.37 ± 0.06	1.86 ± 0.05	2.00 ± 0.01
	2	2.60 ± 0.12	−0.04 ± 0.00	1.47 ± 0.04
	3	2.36 ± 0.16	0.98 ± 0.01	1.26 ± 0.05
	4	1.79 ± 0.03	1.47 ± 0.04	0.90 ± 0.03
	5	1.66 ± 0.06	1.73 ± 0.02	1.32 ± 0.55
3.5	1	1.87 ± 0.02	2.83 ± 0.08	2.75 ± 0.01
	2	1.54 ± 0.00	0.00 ± 0.02	1.31 ± 0.02
	3	0.77 ± 0.09	0.82 ± 0.23	1.01 ± 0.22
	4	1.73 ± 0.00	2.13 ± 0.19	1.80 ± 0.23
	5	2.07 ± 0.00	0.76 ± 0.01	0.76 ± 0.01
4.0	1	1.16 ± 0.06	2.83 ± 0.02	3.06 ± 0.00
	2	0.80 ± 0.06	0.09 ± 0.01	1.49 ± 0.03
	3	1.09 ± 0.05	−0.29 ± 0.17	0.36 ± 0.07
	4	1.69 ± 0.02	1.10 ± 0.03	1.10 ± 0.18
	5	1.93 ± 0.11	1.49 ± 0.01	0.94 ± 0.03

## Data Availability

The data presented in this study are contained within the article and Supplementary Materials.

## Supplementary Materials

The following are available online at https://www.mdpi.com/article/10.3390/s21113817/s1. Figure S1: Statistical difference between the recorded average temperatures for the five volunteers; Table S1: Measured dry and wet forehead temperatures against the distance between 2–4 cm of the five volunteers.
