# Supplementary material for: Design and Development of a Low Cost, Non-Contact Infrared Thermometer with Range Compensation
Source: Sensors (Basel). 2021 May 31;21(11):3817. doi: 10.3390/s21113817 (PMC8198193; doi:10.3390/s21113817)
Supplement: Supplementary file 1 [file sensors-21-03817-s001.zip › sensors-1231292-supplementary (1).pdf]

*Article*

# Design and Development of a Low Cost, Non-Contact Infrared Thermometer with Range Compensation

**Nicholas Wei-Jie Goh [1], Jun-Jie Poh [1], Joshua Yi Yeo [1], Benjamin Jun-Jie Aw [1], Szu Cheng Lai [2], Jayce Jian Wei Cheng [2], Christina Yuan Ling Tan [2] and Samuel Ken-En Gan [1,3,*]**

[1] Antibody & Product Development Lab, EDDC, Agency for Science, Technology and Research (A*STAR), Singapore 138672, Singapore; nicholas_goh@alumni.sutd.edu.sg (N.W.-J.G.); anson_12@hotmail.com (J.-J.P.); joshua_yeo@eddc.a-star.edu.sg (J.Y.Y.); benjaminaw021@gmail.com (B.J.-J.A.)

[2] Institute of Materials Research and Engineering, A*STAR, Singapore 138634, Singapore; sc-lai@imre.a-star.edu.sg (S.C.L.); jayce_cheng@imre.a-star.edu.sg (J.J.W.C.); yl-tan@imre.a-star.edu.sg (C.Y.L.T.)

[3] Department of Psychology, James Cook University, Singapore 387380, Singapore

* Correspondence: samuel_gan@eddc.a-star.edu.sg or samgan@apdskeg.com; Tel.: +65-6407-0584

**Table S1. Measured dry and wet forehead temperature against distance between 2 - 4 cm of the five volunteers.**

| Distance (cm) | Volunteer | Centre | | | | Left | | | | Right | | | |
|---|---|---|---|---|---|---|---|---|---|---|---|---|---|
| | | Dry | Wet | Difference (Dry - Wet) | Sig. (2-tailed) | Dry | Wet | Difference (Dry - Wet) | Sig. (2-tailed) | Dry | Wet | Difference (Dry - Wet) | Sig. (2-tailed) |
| 2.0 | 1 | 34.46 ± 0.02 | 33.13 ± 0.03 | 1.34 ± 0.01 | 0.00** | 30.38 ± 0.01 | 32.23 ± 0.03 | -2.85 ± 0.02 | 0.00** | 34.46 ± 0.02 | 32.90 ± 0.02 | 1.57 ± 0.01 | 0.01* |
| | 2 | 33.08 ± 0.17 | 31.22 ± 0.02 | 1.85 ± 0.08 | 0.02* | 34.30 ± 0.17 | 33.50 ± 0.00 | 0.80 ± 0.07 | 0.10 | 34.75 ± 0.12 | 32.40 ± 0.54 | 2.35 ± 0.25 | 0.06 |
| | 3 | 34.70 ± 0.04 | 32.20 ± 0.06 | 2.49 ± 0.03 | 0.00** | 34.84 ± 0.00 | 32.83 ± 0.13 | 2.01 ± 0.06 | 0.01* | 33.89 ± 0.02 | 33.55 ± 0.10 | 0.34 ± 0.05 | 0.19 |
| | 4 | 34.35 ± 0.07 | 32.45 ± 0.04 | 1.89 ± 0.04 | 0.01* | 33.32 ± 0.01 | 31.91 ± 0.05 | 1.41 ± 0.02 | 0.01* | 32.27 ± 0.43 | 31.31 ± 0.02 | 0.96 ± 0.19 | 0.19 |
| | 5 | 34.47 ± 0.00 | 33.44 ± 0.13 | 1.03 ± 0.06 | 0.05 | 34.67 ± 0.16 | 33.12 ± 0.00 | 1.55 ± 0.07 | 0.02* | 32.74 ± 0.01 | 32.92 ± 0.49 | -0.18 ± 0.22 | 0.73 |
| 2.5 | 1 | 34.35 ± 0.03 | 31.91 ± 0.03 | 1.69 ± 0.05 | 0.00** | 34.11 ± 0.00 | 31.91 ± 0.03 | 2.20 ± 0.01 | 0.00** | 34.40 ± 0.01 | 31.59 ± 0.61 | 2.81 ± 0.27 | 0.04* |
| | 2 | 33.86 ± 0.01 | 32.28 ± 0.01 | 1.58 ± 0.01 | 0.00** | 34.19 ± 0.01 | 33.91 ± 0.03 | 0.28 ± 0.01 | 0.09 | 34.79 ± 0.16 | 34.54 ± 0.03 | 0.26 ± 0.07 | 0.59 |
| | 3 | 34.21 ± 0.00 | 32.14 ± 0.06 | 2.07 ± 0.02 | 0.01* | 34.28 ± 0.02 | 32.73 ± 0.03 | 1.55 ± 0.01 | 0.01* | 32.85 ± 0.25 | 33.20 ± 0.32 | -0.35 ± 0.18 | 0.64 |
| | 4 | 33.98 ± 0.00 | 31.65 ± 0.11 | 2.33 ± 0.05 | 0.01* | 33.65 ± 0.10 | 32.31 ± 0.14 | 1.34 ± 0.08 | 0.02* | 33.62 ± 0.07 | 31.07 ± 0.38 | 2.55 ± 0.17 | 0.04* |
| | 5 | 33.32 ± 1.99 | 32.81 ± 0.00 | 0.51 ± 0.89 | 0.67 | 34.02 ± 0.02 | 33.30 ± 0.00 | 0.72 ± 0.01 | 0.03* | 33.44 ± 0.46 | 33.68 ± 0.21 | -0.24 ± 0.22 | 0.77 |
| 3.0 | 1 | 33.52 ± 0.13 | 32.47 ± 0.11 | 1.37 ± 0.06 | 0.02* | 34.33 ± 0.01 | 32.47 ± 0.11 | 1.86 ± 0.05 | 0.01* | 33.98 ± 0.02 | 31.98 ± 0.00 | 2.00 ± 0.01 | 0.00** |
| | 2 | 33.36 ± 0.01 | 30.76 ± 0.27 | 2.60 ± 0.12 | 0.03* | 33.47 ± 0.01 | 33.51 ± 0.00 | -0.04 ± 0.00 | 0.57 | 34.41 ± 0.08 | 32.94 ± 0.00 | 1.47 ± 0.04 | 0.03* |
| | 3 | 34.13 ± 0.33 | 31.77 ± 0.13 | 2.36 ± 0.16 | 0.01* | 33.94 ± 0.01 | 32.96 ± 0.02 | 0.98 ± 0.01 | 0.00** | 33.52 ± 0.02 | 32.26 ± 0.12 | 1.26 ± 0.05 | 0.05 |
| | 4 | 33.29 ± 0.03 | 31.50 ± 0.06 | 1.79 ± 0.03 | 0.02* | 32.88 ± 0.03 | 31.41 ± 0.08 | 1.47 ± 0.04 | 0.00** | 32.83 ± 0.07 | 31.93 ± 0.01 | 0.90 ± 0.03 | 0.02* |
| | 5 | 33.77 ± 0.14 | 32.11 ± 0.00 | 1.66 ± 0.06 | 0.03* | 34.26 ± 0.04 | 32.54 ± 0.03 | 1.73 ± 0.02 | 0.02* | 33.24 ± 0.05 | 31.92 ± 1.23 | 1.32 ± 0.55 | 0.26 |
| 3.5 | 1 | 33.95 ± 0.05 | 31.93 ± 0.00 | 1.87 ± 0.02 | 0.01* | 34.76 ± 0.18 | 31.93 ± 0.00 | 2.83 ± 0.08 | 0.01* | 34.68 ± 0.02 | 31.93 ± 0.00 | 2.75 ± 0.01 | 0.00** |
| | 2 | 33.78 ± 0.01 | 32.25 ± 0.00 | 1.54 ± 0.00 | 0.00** | 33.86 ± 0.04 | 33.86 ± 0.00 | 0.00 ± 0.02 | 1.00 | 34.75 ± 0.02 | 33.44 ± 0.04 | 1.31 ± 0.02 | 0.00** |
| | 3 | 33.75 ± 0.19 | 32.98 ± 0.04 | 0.77 ± 0.09 | 0.22 | 33.93 ± 0.36 | 33.10 ± 0.37 | 0.82 ± 0.23 | 0.02* | 33.43 ± 0.46 | 32.41 ± 0.19 | 1.01 ± 0.22 | 0.17 |
| | 4 | 34.57 ± 0.01 | 32.84 ± 0.00 | 1.73 ± 0.00 | 0.00** | 33.81 ± 0.01 | 31.68 ± 0.43 | 2.13 ± 0.19 | 0.05 | 33.34 ± 0.50 | 31.54 ± 0.06 | 1.80 ± 0.23 | 0.12 |
| | 5 | 34.17 ± 0.01 | 32.10 ± 0.00 | 2.07 ± 0.00 | 0.00** | 33.36 ± 0.01 | 32.60 ± 0.01 | 0.76 ± 0.01 | 0.02* | 33.33 ± 0.01 | 32.57 ± 0.01 | 0.76 ± 0.01 | 0.02** |
| 4.0 | 1 | 33.09 ± 0.12 | 32.78 ± 0.04 | 1.16 ± 0.06 | 0.04* | 35.61 ± 0.00 | 32.78 ± 0.04 | 2.83 ± 0.02 | 0.00** | 35.29 ± 0.00 | 32.23 ± 0.01 | 3.06 ± 0.00 | 0.00** |
| | 2 | 33.09 ± 0.12 | 32.29 ± 0.02 | 0.80 ± 0.06 | 0.08 | 34.38 ± 0.02 | 34.30 ± 0.01 | 0.09 ± 0.01 | 0.08 | 35.18 ± 0.00 | 33.68 ± 0.08 | 1.49 ± 0.03 | 0.02* |
| | 3 | 34.60 ± 0.11 | 33.51 ± 0.04 | 1.09 ± 0.05 | 0.07 | 33.56 ± 0.37 | 33.86 ± 0.06 | -0.29 ± 0.17 | 0.05 | 32.54 ± 0.03 | 32.18 ± 0.15 | 0.36 ± 0.07 | 0.47 |
| | 4 | 34.32 ± 0.00 | 32.63 ± 0.04 | 1.69 ± 0.02 | 0.01* | 33.50 ± 0.04 | 32.40 ± 0.06 | 1.10 ± 0.03 | 0.00** | 32.92 ± 0.41 | 31.82 ± 0.00 | 1.10 ± 0.18 | 0.13 |
| | 5 | 34.48 ± 0.01 | 32.55 ± 0.24 | 1.93 ± 0.11 | 0.04* | 34.42 ± 0.02 | 32.93 ± 0.01 | 1.49 ± 0.01 | 0.00** | 33.42 ± 0.01 | 32.48 ± 0.08 | 0.94 ± 0.03 | 0.06 |

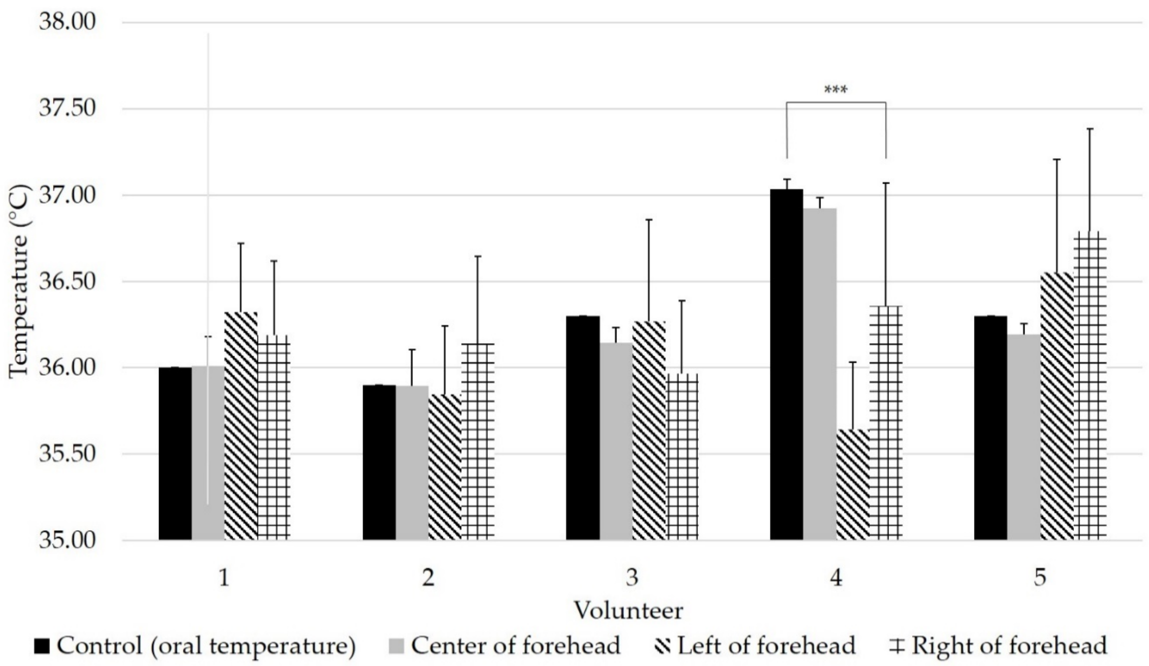


**Figure S1: Statistical difference between the recorded average temperatures for the five volunteers.** *** depicts p-values of statistical tests of the mean difference against the control (oral temperature) at $p < 0.001$.
